# Supplementary material for: Therapeutic Vaccinations with p210 Peptides in Imatinib-Treated Chronic Myeloid Leukemia Patients: 10 Years Follow-Up of GIMEMA CML0206 and SI0207 Studies
Source: Vaccines (Basel). 2025 Apr 16;13(4):419. doi: 10.3390/vaccines13040419 (PMC12031404; doi:10.3390/vaccines13040419)
Supplement: Supplementary file 1 [file vaccines-13-00419-s001.zip › vaccines-3549317-supplementary.pdf]

Supplementary Material and Methods

Amino acid sequences included in each peptide vaccine

CMLVAX100

The vaccine consisted of 100µg per each of five b3a2 breakpoint-derived peptides: ATGFKQSSK, A11-binding; KQSSKALQR, A3-binding; HSATGFKQSSK A3 and A11-binding; GFKQSSKAL B8-binding and 25 amino acid peptide IVHSATGFKQSSKALQRPVASDFEP with binding motifs for HLA class II DR1, DR4, DR15 and DR11.

CMLVAXb2a2-25

The vaccine consisted of one single 25 amino acid long peptide TVHSIPLTINKEEALQRPVASDFEP with binding capacity for HLA DR1, DR2, DR3, DR4, DR7, DR11, DR15, DR17. The inclusion of only 1 HLA class II binder b2a2 breakpoint derived peptide into CMLVAXb2a2-25 vaccine (SI0207 trial) was due to the lack of identification of short HLA Class I binders b2a2-breakpoint derived peptides during pre-clinical studies.

Peptide vaccine manufacturing information

CMLVAX100

The vaccine batch n° 1-05 of CMLVAX100 has been prepared in house in accordance with GMP standards equivalent to those laid down in Directive 91/356/EEC (DL 29/5/91 n°178) as requested for product manufactured in EU without marketing authorization according to the guidelines described in the "European Commission VOLUME 4 Good manufacturing practices ANNEX 13 manufacture of investigational medicinal products July 2003". The production process has followed exactly the procedure previously approved by the Istituto Superiore di Sanità (ISS) (*1/12/1998 N° 34060 DE 21-776; Sper /0.40.1/ 2917)*.

CMLVAXb2a2-25

This investigational medicinal product has been produced by the company Bachem Distribution Services GmbH (BDS) Hegenheimer Strasse 5 79576 Weil am Rhein Germany. BDS was responsible for manufacture, analytical testing for sterility and endotoxin content and release as well as storage and shipment of the b2a2-25 100 µg vials. CMLVAXb2a2-25 has been approved by the Istituto Superiore di Sanità (ISS) (N° 53374-PRE21-864; Prot.19/09/2006-0046796) for the use in b2a2-CML patients.

CD4+ T cells purification assay

CD4+T cells purification has been performed by using a magnetic separation with microbeads (Miltenyi Biotec). Briefly, after a separation of peripheral blood mononuclear cells (PBMCs) cells with a density gradient solution (Ficoll-Paque), the CD4+ cells have been magnetically labeled with CD4 Microbeads (20µl x 10^7 total cells). The cell suspension has been incubated for 15 minutes in the refrigerator (2-8°C) and centrifuged at 300xg for 10 minutes. Then, the cells have been resuspended in 500 µl of buffer solution containing phosphate-buffered saline (PBS) and loaded on a column which is placed in the magnetic field of a separator. The magnetically labeled CD4+ cells have been retained within the column and after removing the column

from the magnetic field, the magnetically retained CD4+ cells have been eluted as the positively selected cell fraction.

BCR::ABL1 transcript monitoring

Molecular evaluations of BCR::ABL1 transcript have been performed according to the standardized procedures. Briefly, peripheral blood samples from CML patients have been centrifuged at 200xg for 10 minutes to obtain the buffy coat from which the mRNA has been extracted. After the isolation of mRNA, a complementary DNA (cDNA) has been obtained by reverse transcription process.

Then, reverse transcriptase quantitative PCR (RT-qPCR) has been performed to estimate the amount of BCR::ABL1 relative to an internal reference gene, most commonly ABL1, or GUSB. The results were expressed on an International Scale (IS) as a percentage. Expression of results on the IS depends on each testing laboratory either having obtained a laboratory-specific conversion factor (CF) by sample exchange with an established reference laboratory or by using kits and reagents that have been calibrated to the World Health Organization International Genetic Reference Panel for quantitation of BCR::ABL1 mRNA. Specifically, results have been expressed as the *BCR::ABL1/ABL1* % IS value: (*BCR::ABL1/ABL1* ratio) x 100 x conversion factor.
